# Supplementary material for: Splice-Junction-Based Mapping of Alternative Isoforms in the Human Proteome
Source: Cell Rep. Author manuscript; Available in PMC 2020 Jan 15. (PMC6961840; doi:10.1016/j.celrep.2019.11.026)
Supplement: 2 [file NIHMS1546469-supplement-2.zip › DF1/PXD006675/RightCoronaryArtery/RightCoronaryArtery_12_SPTAN1_HQWIQETRSCMVEESGTLESQLEATK.pdf]

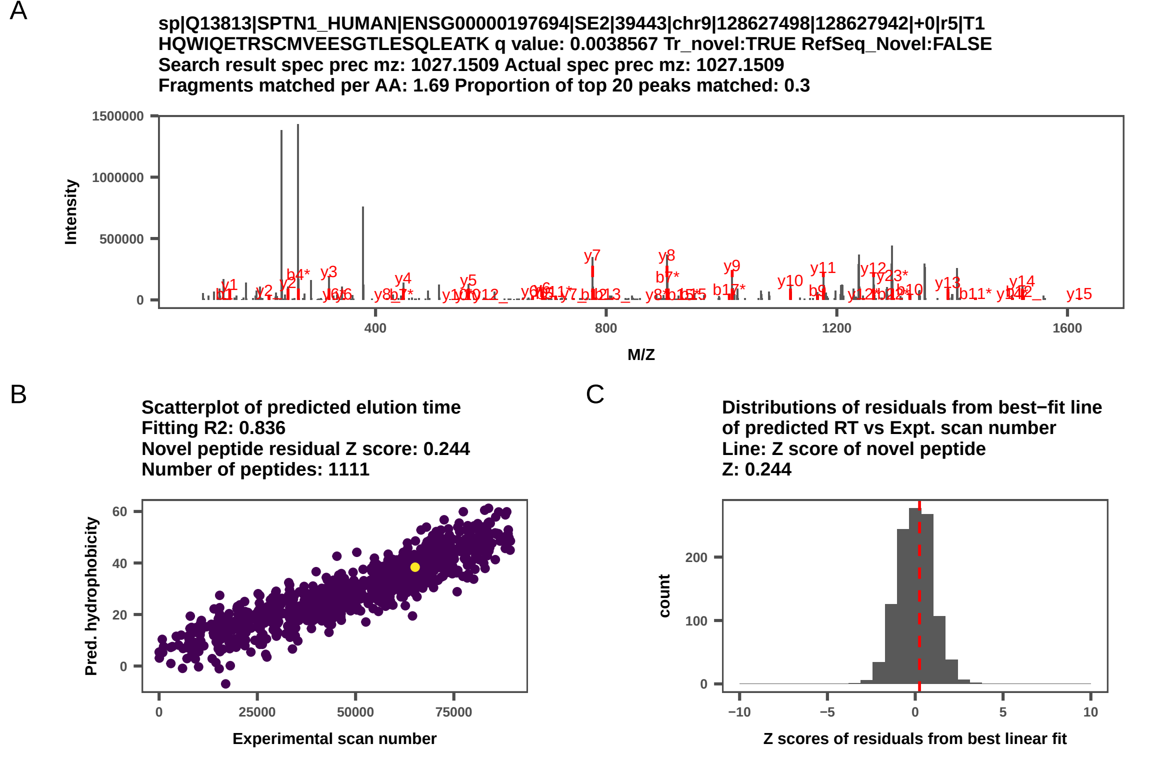
A
sp|Q13813|SPTN1_HUMAN|ENSG00000197694|SE2|39443|chr9|128627498|128627942|+0|r5|T1
HQWIQETRSCMVEESGTLESQLEATK q value: 0.0038567 Tr_novel:TRUE RefSeq_Novel:FALSE
Search result spec prec mz: 1027.1509 Actual spec prec mz: 1027.1509
Fragments matched per AA: 1.69 Proportion of top 20 peaks matched: 0.3
Intensity
1500000
1000000
500000
0
y7
y8
y9
y11
y12
b4*
y3
y4
y5
y10
y13
y14
y15
M/Z
400
800
1200
1600
B
Scatterplot of predicted elution time
Fitting R2: 0.836
Novel peptide residual Z score: 0.244
Number of peptides: 1111
Pred. hydrophobicity
60
40
20
0
25000
50000
75000
Experimental scan number
C
Distributions of residuals from best−fit line
of predicted RT vs Expt. scan number
Line: Z score of novel peptide
Z: 0.244
count
200
100
0
−10
−5
0
5
10
Z scores of residuals from best linear fit
